# Autoimmune Liver Disease: News and Perspectives

**Published:** 2011-10-17

**Authors:** Giorgina Mieli-Vergani, Diego Vergani

**Affiliations:** King’s College London School of Medicine at King’s College Hospital, Denmark Hill, London SE5 9RS, UK

**Keywords:** autoimmune hepatitis, autoimmune sclerosing cholangitis, corticosteroids, azathioprine, calcineurin inhibitors, mycophenolate mofetil, ursodeoxycholic acid, regulatory T-cells

## Abstract

Autoimmune liver disorders in childhood include autoimmune hepatitis and autoimmune sclerosing cholangitis. These inflammatory liver disorders are characterised histologically by interface hepatitis, biochemically by elevated transaminase levels and serologically by autoantibodies and increased levels of immunoglobulin G. Autoimmune hepatitis is particularly aggressive in children and progresses rapidly unless immunosuppressive treatment is started promptly. With appropriate treatment 80% of patients achieve remission and long-term survival. Autoimmune sclerosing cholangitis responds to the same treatment used for autoimmune hepatitis in regards to parenchymal inflammation, but bile duct disease progresses in about 50% of cases, leading to a worse prognosis and higher transplantation requirement; it has a high recurrence rate post liver transplant. New strategies aiming at treating unresponsive patients and at curbing specifically the liver autoimmune attack, without provoking unwanted systemic side effects, are being investigated and may be available within the next 5 years.

## Introduction

Autoimmune liver diseases (AILD) are important in the differential diagnosis of liver disease in childhood. Their presentation is non-specific and can mimic most other liver disorders. As prompt treatment is life saving, it is imperative to suspect AILD and perform appropriate investigations in all children who present with a cryptogenic liver disorder. Juvenile AILD encompasses autoimmune hepatitis (AIH) (1–2) and autoimmune sclerosing cholangitis (ASC) (3).

## Autoimmune hepatitis

Autoimmune hepatitis (AIH) is a progressive inflammatory liver disorder affecting mainly females, characterized serologically by high levels of transaminases and immunoglobulin G (IgG), and presence of autoantibodies, and histologically by interface hepatitis, in the absence of a known etiology (1–2).

AIH is divided into two types according to the autoantibody profile: type 1 is positive for anti-nuclear (ANA) and/or anti-smooth muscle (SMA) antibody, type 2 is positive for anti-liver kidney microsomal antibody type 1 (anti-LKM-1).

AIH responds satisfactorily to immunosuppressive treatment. If left untreated, it generally progresses rapidly to cirrhosis and liver failure.

The peak incidence of the disease is before puberty and 75% of the patients are girls. The epidemiology of childhood AIH is unknown, but type 1 AIH accounts for two thirds of the cases and presents usually during adolescence, while type 2 AIH presents at a younger age and also during infancy. IgG are usually raised at presentation in both types, though 15% of children with AIH type 1 and 25% of those with AIH type 2 have normal levels (4). IgA deficiency is common in AIH type 2 (4). Severity of disease is similar in the two types. Anti-LKM-1 positive patients, however, have a higher tendency to present as acute liver failure. Both types are often associated with autoimmune disorders (about 20%) and a family history of autoimmune disease (40%) (4). Type 2 AIH can be part of the autoimmune polyendocrinopathy-candidiasis-ectodermal dystrophy (APECED) syndrome, an autosomal recessive genetic disorder in which the liver disease is reportedly present in 20% of cases (5).

## Autoimmune sclerosing cholangitis

In pediatrics, primary sclerosing cholangitis is often associated with florid autoimmune features, including elevated titers of autoantibodies, in particular ANA and SMA, elevated IgG, and interface hepatitis (3). Since these features are shared in common with AIH and are often not accompanied by elevated alkaline phosphatase or gamma glutamyl transpeptidase levels at disease onset, the diagnosis of sclerosing cholangitis relies on cholangiographic studies. In the absence of cholangiographic studies at presentation many of these children are diagnosed and treated as AIH, though the diagnosis of sclerosing cholangitis may become apparent during follow up. This condition, named autoimmune sclerosing cholangitis, (ASC), is as prevalent as AIH type 1 in childhood, but in contrast to AIH it affects equally boys and girls (3). ASC responds satisfactorily to immunosuppression, at least in regard to the parenchymal inflammation, if treatment is started early, but its medium to long term prognosis is worse than that of AIH because of progression of bile duct disease in some 50% of patients (3).

## Diagnostic criteria

**Figure 1:**
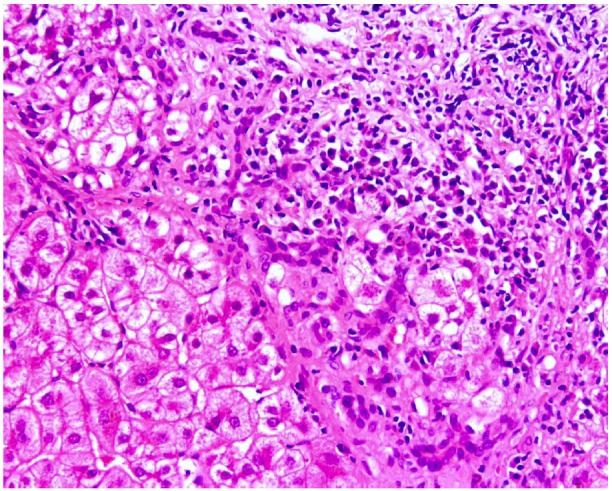
Interface hepatitis: portal and periportal lymphocyte and plasma cell infiltrate extending to and disrupting the parenchymal limiting plate (haematoxylin & eosin, original magnification x20). (Figure kindly provided by Dr. Alex Knisely)

Diagnosis of AIH is based on a series of positive and negative criteria (6), (7). Liver biopsy is necessary to establish the diagnosis, the typical histological picture including a dense mononuclear and plasma cell infiltration of the portal areas, which expands into the liver lobule; destruction of the hepatocytes at the periphery of the lobule with erosion of the limiting plate (‘interface hepatitis’) (**Figure 1**); connective tissue collapse resulting from hepatocyte death and expanding from the portal area into the lobule (‘bridging collapse’); hepatic regeneration with ‘rosette’ formation. In addition to the typical histology, other positive criteria include elevated serum transaminase and IgG levels, and presence of ANA, SMA or anti-LKM-1.

The diagnosis of AIH has been advanced by the criteria developed by the International Autoimmune Hepatitis Group (IAIHG) for adult patients (6), (7) where negative criteria such as evidence of infection with hepatitis B or C virus or Wilson disease, alcohol, among others, are taken into account in addition to the positive criteria mentioned above. The IAIHG scoring system was devised mainly for research purposes to allow ready comparison between series from different centres, but has also been used clinically, including in paediatric series (8), (4).

**Figure 2:**
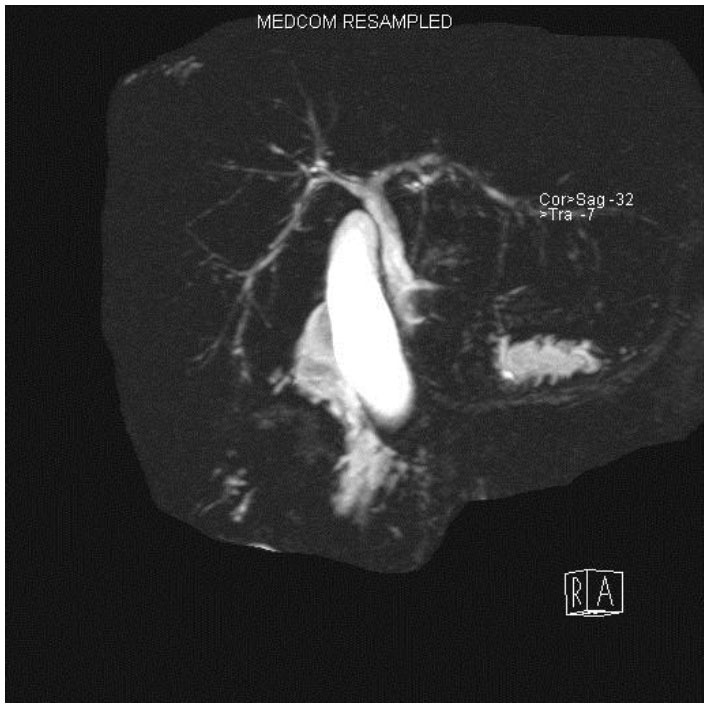
Magnetic resonance cholangiography of a child with autoimmune sclerosing cholangitis showing diffuse intrahepatic cholangiopathy affecting both liver lobes. (Figure kindly provided by Dr. Maria Sellars)

More recently the IAIHG have published a simplified scoring system based on autoantibodies, IgG, histology, and exclusion of viral hepatitis that is better suited to clinical application (9). However, neither scoring system is perfect, in particular for the juvenile form of the disease, where diagnostically relevant autoantibodies often have titres lower than the cut-off value considered positive in adults. Thus, immunofluorescence titres of 1:20 for ANA and SMA and of 1:10 for anti-LKM-1 are significant in paediatrics. In addition, neither system can distinguish between AIH and ASC (10), which can only be differentiated if a cholangiogram using magnetic resonance or retrograde cholangiograpahy is performed at disease presentation (3) (**Figure 2**).

**Figure 3:**
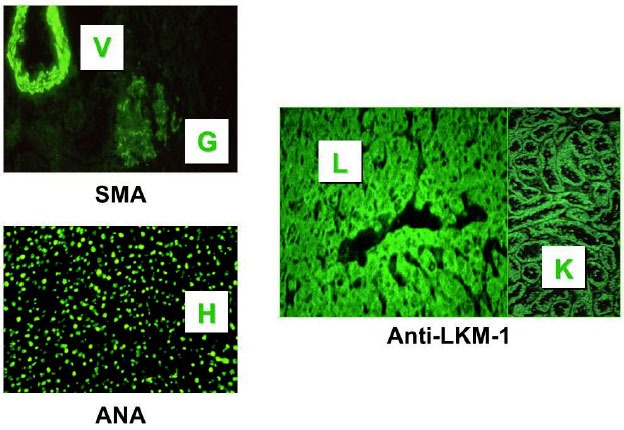
Immunofluorescence pattern of anti-nuclear antibody (ANA), smooth muscle antibody (SMA) and anti-liver kidney microsomal type 1 antibody. In autoimmune hepatitis and autoimmune sclerosing cholangitis ANA usually has a homogeneous pattern (H); SMA stains the smooth muscle of arterial vessels (V), glomeruli (G), and tubules (not shown). Anti-LKM-1, which characterizes autoimmune hepatitis type 2, stains the cytoplasm of hepatocytes (H) and proximal renal tubules (R)

A key diagnostic criterion for autoimmune liver disease is the detection of autoantibodies to nuclei (ANA), smooth muscle (SMA) and liver kidney microsomes (anti-LKM-1) (**Figure 3**). Autoantibody detection, which should be made using rat liver, kidney and stomach as substrate in indirect immunofluorescence, not only assists in the diagnosis but also allows differentiation of AIH types. ANA and SMA characterize type 1 AIH and ASC; anti-LKM-1 defines type 2 AIH. In the rare instances when ANA and/or SMA are present simultaneously with anti-LKM-1, the clinical course is similar to that of AIH type 2 (11).

Positivity for autoantibodies, however, is not sufficient for the diagnosis of AILD as they can be present, usually at low titre, in other liver disorders such as viral hepatitides (12), (13), Wilson disease (14) and non alcoholic steatohepatitis (15).

Other autoantibodies less commonly tested but of diagnostic importance include anti liver cytosol type 1 (LC-1), peripheral anti nuclear neutrophil antibody (atypical pANCA or pANNA) and anti soluble liver antigen (SLA). Anti-LC-1, which can be present on its own, but frequently occurs in association with anti-LKM-1, is an additional marker for AIH type 2 (16). pANNA is frequently found in ASC and AIH type 1, and is also common in inflammatory bowel disease, while it is virtually absent in type 2 AIH. Anti-SLA, originally described as the hallmark of a third type of AIH (17), is also found in 50% of patients with type 1 and type 2 AIH, where it defines a more severe course (18).

The criteria for the diagnosis of autoimmune liver disease in childhood are summarised in the Table.

**Table.** **Criteria for the diagnosis of autoimmune liver disease in childhood**

• Elevated transaminases	
• Positive autoantibodies:	ANA and/or SMA (titre ≥ 1:20) = AIH-1 or ASC Anti-LKM1 (titre ≥ 1:10) = AIH-2 Anti-LC1 = AIH-2 Anti-SLA = present in AIH-1, AIH-2, ASC or in isolation
• Elevate immunoglobulin G	∼ 80% of cases
• Liver biopsy:	interface hepatitis multilobular collapse
• Exclusion of viral hepatitis	
• Exclusion of Wilson disease	
• Exclusion of non alcoholic steatohepatitis	
• Normal cholangiogram	AIH
• Abnormal cholangiogram	ASC

AIH, autoimmune hepatitis; ASC, autoimmune sclerosing cholangitis; ANA, anti-nuclear antibodies; SMA, anti-smooth muscle antibody; anti-LKM1, anti-liver kidney microsomal type 1 antibody; anti-LC1, anti-liver cytosol type 1 antibody; anti-SLA, anti-soluble liver antigen antibody

There is a small proportion of patients with all the features of AIH, but without detectable autoantibodies. This condition, which responds to immunosuppression like the sero-positive form, represents sero-negative AIH (19), a rare form of AIH in adults, whose prevalence and clinical characteristics remain to be defined in children.

## Treatment of autoimmune hepatitis

### Definition of remission/relapse:

Remission is defined as complete clinical recovery, normal transaminase and IgG levels, negative or very low titer autoantibodies by immunofluorescence (≤1:20 for ANA and SMA; ≤1:10 for anti-LKM-1) and histological resolution of inflammation. The histological response lags behind the biochemical response (20) and clinical/biochemical remission does not necessarily reflect histological resolution. Relapse is characterized by increase of serum aminotransferase levels after remission has been achieved. Relapse during treatment is common, occurring in about 40% of patients and requiring a temporary increase in the steroid dose. An important role in relapse is played by non-adherence, particularly in adolescents (21). In more aggressive cases, the risk of relapse is higher if steroids are administered on an alternate-day schedule, which is often instituted in the belief that it has a less negative effect on the child’s growth. Small daily doses are more effective in maintaining disease control and minimize the need for high-dose steroid pulses during relapses (with the consequent more severe side effects) and do not affect final height (22).

### When to treat:

AIH should be suspected and sought in all children with evidence of liver disease after exclusion of infectious and metabolic aetiologies. AIH is exquisitely responsive to immunosuppression and treatment should be initiated promptly to avoid progression of disease. The goal of treatment is to reduce or eliminate liver inflammation, to induce remission, improve symptoms, and prolong survival (2). The rapidity and degree of the response depends on the disease severity at presentation. Although cirrhosis is found between 44% and 80% of children at the time at diagnosis, (4), (23) mortality is low and most children remain clinically stable, with a good quality of life on long-term treatment.

### How to treat:

With the exception of a fulminant presentation with encephalopathy, where liver transplant is usually required, AIH responds satisfactorily to immunosuppressive treatment whatever the degree of liver impairment, with a reported remission rate exceeding 80%.

### Standard treatment:

Conventional treatment of AIH consists of prednisolone (or prednisone) 2 mg/kg/day (maximum 40–60 mg/day), which is gradually decreased over a period of 4–8 weeks, in parallel to the decline of transaminase levels, to a maintenance dose of 2.5–5 mg/day (24) (2). In most patients an 80% decrease of the aminotransferase levels is achieved in the first two months, but their complete normalization may take several months (24), (25) . During the first 6–8 weeks of treatment, liver function tests should be checked frequently to allow weekly dose adjustments, avoiding severe steroid side effects. In our centre, azathioprine is added as a steroid sparing agent if the transaminase levels stop decreasing on steroid treatment alone or in the presence of early serious steroid side effects (e.g. psychosis), at a starting dose of 0.5 mg/kg/day, which in the absence of signs of toxicity is increased up to a maximum of 2.0–2.5 mg/kg/day until biochemical control is achieved. The timing for the addition of azathioprine varies in different centres. In some azathioprine is added at a dose of 0.5–2 mg/kg/day after a few weeks of steroid treatment. Other centres use a combination of steroids and azathioprine from the beginning, but caution is recommended because azathioprine can be hepatotoxic, particularly in jaundiced patients. Whatever the protocol, 85% of the patients eventually require the addition of azathioprine.

Measurement of thiopurine methyltransferase activity level before initiating azathioprine therapy has been advocated to predict azathioprine metabolism and toxicity. However, only patients with near-zero erythrocyte concentrations of thiopurine methyltransferase activity are at risk for myelosuppression during azathioprine treatment (26), and determination of the enzyme activity is warranted only when there is pre- or intra-treatment cytopaenia, or the need of higher than conventional doses (27). Measurement of the azathioprine metabolites 6-thioguanine and 6-methylmercaptopurine has been reported to help in identifying drug toxicity and non adherence and in achieving a level of 6-thioguanine considered therapeutic for inflammatory bowel disease (28), though an ideal therapeutic level for AIH has not been determined.

### Alternative treatment:

Induction of remission has been obtained in treatment naïve children using cyclosporine A alone for 6 months, followed by the addition of prednisone and azathioprine; one month later the cyclosporine is discontinued (29), (30). Cyclosporine was used at the dose of 4 mg/kg/day in three divided doses, increased if necessary every 2 to 3 days to achieve a whole blood concentration of 250±50 ng/ml for 3 months. If there was clinical and biochemical response in the first months, cyclosporine was reduced to achieve a concentration of 200±50 ng/ml for the following 3 months, before discontinuing it. Whether this mode of induction has any advantage over the standard treatment has yet to be evaluated in controlled studies.

Tacrolimus is a more potent immunosuppressive agent than cyclosporine, but it also has significant toxicity. There is limited evidence supporting its role as primary treatment in AIH apart from anecdotal reports in adults.

Budesonide has a hepatic first-pass clearance of > 90% of oral dose and less side effects than predniso(lo)ne, but cannot be used in cirrhotic patients, who represent a large proportion of AIH patients. In a large European study, a combination of budesonide and azathioprine had fewer adverse effects compared to medium-dose standard prednisone and azathioprine (31). In this study, budesonide at a dose of 3 mg three times daily, decreased upon response, was compared with prednisone 40 mg once daily reduced per protocol irrespective of response. After 6 months of treatment, remission was achieved in 60% of the budesonide group but in only 39% of the prednisone group, both percentages being worse than those achieved with standard treatment (4). The results among the children recruited into this study were particularly disappointing, with a similarly low remission rate of 15.8% for budesonide and 14.8% for prednisone after 6 months of treatment and of 50% and 41.7% respectively after 12 months of treatment (32). Nevertheless, budesonide could be a valid alternative in selected non cirrhotic patients who are at risk of adverse effects from steroids.

### Treatment of refractory cases:

Mycophenolate mofetil (MMF) is the prodrug of mycophenolic acid. Its effect on purine synthesis leads to decreased T and B lymphocyte proliferation. In patients (up to 10%) in whom standard immunosuppression is unable to induce stable remission, or who are intolerant to azathioprine, MMF at a dose of 20 mg/kg twice daily (total daily dose 40 mg/Kg), together with predniso(lo)ne, is successfully used (33). If there is a persistent absence of response or if there is intolerance for MMF (headache, diarrhoea, nausea, dizziness, hair loss, and neutropaenia), the use of calcineurin inhibitors should be considered. Tacrolimus in particular may be useful in combination with predniso(lo)ne as second-line therapy.

#### Treatment of autoimmune sclerosing cholangitis

ASC responds to the same immunosuppressive treatment described above for AIH. However, while steroids and azathioprine are beneficial in abating the parenchymal inflammatory lesions, they appear to be less effective in controlling the bile duct disease. Ursodeoxycholic acid (UDCA) is usually added to steroids and azathioprine for the treatment of ASC, but whether it is helpful in arresting the progression of the bile duct disease remains to be established. In adults with primary sclerosing cholangitis high-dose UDCA has been reported as more beneficial than standard doses (34), but a randomized double-blind controlled study presented from the Mayo Clinic shows that high-dose UDCA has a negative long-term effect (35). It is prudent, therefore, to use doses not higher than 15–20 mg/Kg/day. ASC is often associated with inflammatory bowel disease which should be investigated even in the absence of symptoms and appropriately treated.

## Duration of treatment and prognosis

The optimal duration of immunosuppressive treatment for AILD is unknown. Treatment withdrawal is successful only if there is histological resolution of inflammation. Hence, cessation of treatment should considered if a liver biopsy shows minimal or no inflammatory changes after 1–2 years of normal liver function tests, normal IgG levels and negative or low titre autoantibodies. However, it is advisable not to attempt to withdraw treatment within 3 years of diagnosis or during or immediately before puberty, when relapses are more common. It has been reported that 20% of patients with AIH type 1 and some 10% of those with ASC can successfully and permanently stop treatment, while this is rarely achieved in AIH type 2 (3–4). Long term treatment is required for the majority of patients and parents and patients should be counselled accordingly. In the paediatric setting, an important role in monitoring the response to treatment is the measurement of IgG levels and autoantibody titres, the fluctuation of which correlates with disease activity (36). In particular, for patients with high IgG levels, their decrease is a reliable, objective and inexpensive measure of disease control.

The prognosis of children with AIH who respond to immunosuppressive treatment is generally good, with most patients surviving long-term with excellent quality of life on low dose medication. Development of end-stage liver disease requiring liver transplantation despite treatment, however, has been reported 8–14 years after diagnosis in 8.5% of children with AI (4). The prognosis of children with ASC is worse, as bile duct disease progresses despite treatment in some 50% (3).

## Liver transplantation

Liver transplantation is indicated in patients with AILD who present with fulminant hepatic failure (with encephalopathy) and those who develop end-stage liver disease. The latter is more likely when established cirrhosis is present at diagnosis, or if there is a long history of liver disease before the start of treatment. Approximately 10% of children with AIH and 20% of those with ASC require liver transplantation. After transplantation, recurrent AIH has been described in about 20% of cases (37) and recurrent ASC in about 70% (38). Diagnosis of recurrence is based on biochemical abnormalities, presence of autoantibodies, interface hepatitis on liver histology, steroid dependence, and, for ASC, presence of cholangiopathy. Recurrence may appear even years after transplantation, and consequently maintenance of steroid-based immunosuppression at a higher dose than that used for patients not transplanted for AILD is generally recommended. Additionally, a form of graft dysfunction called de novo AIH, associated with positive autoantibodies, high IgG, histological features of interface hepatitis, and responsiveness to the standard treatment for AIH (but not to anti-rejection regimens) has been described in 6–10% of children transplanted for non autoimmune disorders (39), (40).

## Pathogenesis and animal models

Research on the pathogenesis of AIH has been hampered by the lack of animal models reproducing faithfully the human condition.

A widely studied model of experimental hepatitis is that induced by concanavalin A. Though this model does not reflect accurately the pathological entity of AIH in humans, it has provided evidence that liver damage mainly occurs within a Th1 scenario, with the involvement of activated CD4+ T cells and release of the proinflammatory cytokines interferon gamma and tumour necrosis factor alpha against a specific genetic background. Interleukin-4, a cytokine with mainly regulatory activity, is also required for the establishment of concanavalin A-induced hepatitis. This finding and those of Takeda and collaborators, who have shown that NKT cells, which secrete both interleukin-4 and interferon gamma, are critical to the development of concanavalin A-induced hepatitis in C57/B6 mice, suggest that both adaptive and innate immunity are involved (41, 42).

The model described above, though informative regarding single steps leading to liver inflammation and damage, does not mimic the chronic relapsing course of human AIH. The ideal model for AIH should have a well-defined initiating event followed by chronic inflammation leading to fibrosis. More recently, researchers have focused on animal models of AIH-2, since in this condition the autoantigens are well defined. The model produced by the group of Alvarez (43) is based on immunising every two weeks for three times C57BL/6 female mice with a plasmid containing the antigenic region of human CYP2D6, the target of anti-LKM-1, and FTCD, the target of anti-LC-1, together with the murine end terminal region of cytotoxic T lymphocyte antigen 4 (CTLA-4). The latter was added to facilitate antigen uptake by antigen presenting cells. In a parallel set of experiments a plasmid containing the DNA encoding interleukin-12, a Th1 skewing pro-inflammatory cytokine, was also used.

When autoantigens and interleukin-12 were used to break tolerance, antigen specific autoantibodies were produced, a relatively modest elevation of transaminase levels at 4 and 7 months was observed, and a portal and periportal inflammatory infiltrate composed of CD4 and CD8 T-cells and, to a lesser extent, B cells was demonstrated 8–10 months after the third immunization. When the same immunization protocol was used in different mouse strains, either a mild hepatitis or no inflammatory changes were observed indicating the importance of a specific genetic background. Another model AIH-2 uses CYP2D6 transgenic mice and aims at breaking tolerance with an Adenovirus-CYP2D6 vector (44). While focal hepatocyte necrosis was seen in both mice treated with the Adenovirus-CYP2D6 vector and control mice treated with Adenovirus alone, only the former developed chronic histological changes, including fibrosis, reminiscent of AIH. The hepatic lesion was associated to a specific immune response to an immunodominant region of CYP2D6 and a cytotoxic T-cell response to Adenovirus-CYP2D6 vector infected target cells. Though these two experimental approaches provide useful information on the possible pathogenic mechanisms leading to AIH-2, a model mimicking closely AIH in humans is still missing.

The aetiology of human AILD is unknown, although both genetic and environmental factors are involved (1) (25) (45). The histological hallmark of AILD is a dense portal mononuclear cell infiltrate that invades the surrounding parenchyma (Figure 1) and comprises T and B lymphocytes, macrophages, and plasma cells. An unknown, but powerful stimulus must be promoting the formation of this massive inflammatory cellular reaction that is likely to initiate and perpetuate liver damage.

**Figure 4:**
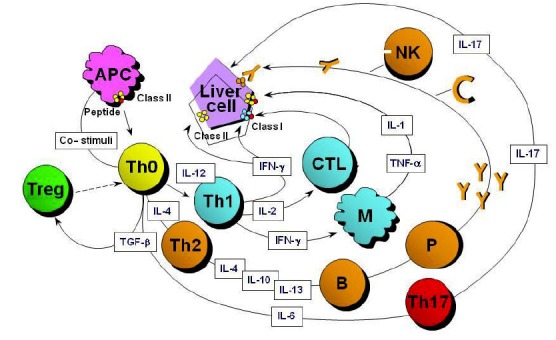
Liver damage in autoimmune liver disease is likely to be orchestrated by CD4 T lymphocytes recognizing an autoantigenic liver peptide. To trigger an autoimmune response, the peptide must be embraced by an HLA class II molecule and presented to naïve CD4 T-helper (Th0) cells by professional antigen-presenting cells (APC), with the co-stimulation of ligand-ligand fostering interaction between the two cells. As Th0 cells become activated, they differentiate into functional phenotypes according to the cytokines prevailing in the microenvironment and the nature of the antigen, and initiate a cascade of immune reactions determined by the cytokines produced by the activated T cells. T-helper type 1 (Th1) cells, arising in the presence of the macrophage-produced interleukin (IL)-12, secrete mainly IL-2 and interferon gamma (IFN-γ), which enhance expression of HLA class I (increasing liver cell vulnerability to a CD8 T cell cytotoxic attack), induce expression of HLA class II molecules on hepatocytes, and activate macrophages, which release the proinflammatory cytokine tumour necrosis factor alpha (TNF-α) and IL-1. T-helper type 2 (Th2) cells, which differentiate from Th0 if the microenvironment is rich in IL-4, produce mainly IL-4, IL-10, and IL-13, which favour autoantibody production by B lymphocytes and plasma cells (P). Autoantibody coated hepatocytes can recruit natural killer (NK) cells and complement (C) as effectors of damage. Physiologically, Th1 and Th2 antagonize each other. In addition, Th17 cells, a recently described population, arise in the presence of transforming growth factor beta (TGF-β) and IL-6 and appear to have an important effector role in inflammation and autoimmunity. The process of autoantigen recognition is strictly controlled by regulatory mechanisms, such as those exerted by CD4+CD25+ regulatory T cells (Treg), which derive from Th0 in the presence of TGF-β, but in the absence of IL-6. If regulatory mechanisms fail, the autoimmune attack is perpetuated.

An autoimmune attack can follow different pathways to inflict damage on hepatocytes (**Figure 4**).

Over the past three decades, different aspects of the pathogenic scenario depicted in **Figure 4** have been investigated (45). Most important for its therapeutic implications (see below), a defect in immunoregulation affecting regulatory T-cells has been demonstrated in AILD, particularly at diagnosis or during relapse.

## Future treatment options

The development of new immunosuppressive agents effective in the patients who fail to respond to conventional corticosteroid treatment, and which may promote permanent resolution of the disease in all patients, is the ultimate goal for AILD.

The armamentarium available to manipulate the immune system in the field of organ transplantation and in other autoimmune diseases may ultimately provide important information for the treatment of AILD. Immunosuppressant medications that may theoretically be useful include selective monoclonal antibodies directed against the IL-2 receptor, a high number of activated lymphocytes bearing this receptor being characteristic of the active phase of the disease (46). However, regulatory T cells also express IL-2 receptor and a fine balance will need to be found between curbing effector function while avoiding interfering with regulatory mechanisms. In view of the elevated level of IgG and high titres of autoantibodies, rituximab is a possible mode of treatment for particularly aggressive cases.

Rapamycin, reportedly successful in the control of post transplant de novo AIH (47), could also have a role in difficult-to-treat cases.

All the above drugs, however, in common with conventional immunosuppressive drugs, do not suppress only the autoimmune process causing liver damage, but also weaken the physiological immune responses, with consequent systemic side effects.

**Figure 5:**
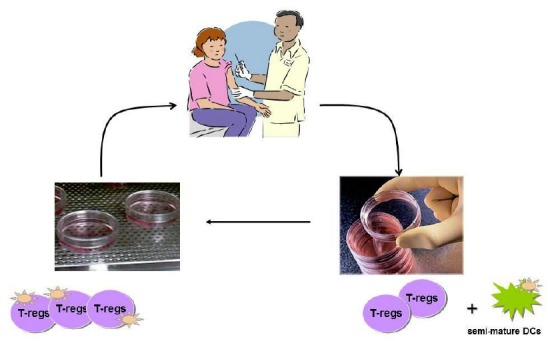
Adoptive transfer of regulatory T-cells (T-regs): blood is drawn from a patient with autoimmune liver disease and T-regs are expanded in the presence of semi-mature dendritic cells pulsed with an autoantigenic peptide. T-regs generated in large quantities under good manufacturing practice (GMP) are then re-infused in the same patient with the aim of reconstituting immune regulatory function and curing the disease (cartoon kindly provided by Dr Maria Serena Longhi)

Recent studies showing that a decrease in number and function of regulatory T cells (T-regs) characterises AILD, particularly when the disease is active, and that defective liver antigen-specific T-regs can be cultured, re-educated and expanded in vitro (48), lay the foundation for treatment based on adoptive transfer of re-educated antigen specific T-regs (**Figure 5**). This would be able to treat, possibly cure, liver directed autoimmunity without impairing the overall function of the immune system.
